# Vectorial Image Representation for Image Classification

**DOI:** 10.3390/jimaging10020048

**Published:** 2024-02-13

**Authors:** Maria-Eugenia Sánchez-Morales, José-Trinidad Guillen-Bonilla, Héctor Guillen-Bonilla, Alex Guillen-Bonilla, Jorge Aguilar-Santiago, Maricela Jiménez-Rodríguez

**Affiliations:** 1 Departamento de Ciencias Tecnológicas, Centro Universitario de la Ciénega, Universidad de Guadalajara, Av. Universidad No. 1115, Lindavista, Ocotlán 47810, Jalisco, Mexico; eugenia.sanchez@academicos.udg.mx; 2 Departamento de Electro-Fotónica, Centro Universitario de Ciencias Exactas e Ingenierías, Universidad de Guadalajara, Blvd. M. García Barragán 1421, Guadalajara 44410, Jalisco, Mexico; 3 Departamento de Ingeniería de Proyectos, Centro Universitario de Ciencias Exactas e Ingenierías, Universidad de Guadalajara, Blvd-M. García Barragán 1421, Guadalajara 44410, Jalisco, Mexico; hector.guillen1775@academicos.udg.mx; 4 Departamento de Ciencias Computacionales e Ingenierías, Centro Universitario de los Valles, Universidad de Guadalajara, Carretera Guadalajara-Ameca Km. 45.5, Ameca 46600, Jalisco, Mexico; alex.guillen@academicos.udg.mx; 5 Departamento de Ciencias Básicas, Centro Universitario de la Ciénega, Universidad de Guadalajara, Av. Universidad No. 1115, Lindavista, Ocotlán 47810, Jalisco, Mexico; jorge.asantiago@academicos.udg.mx

**Keywords:** Vectorial Image Representation on the Texture Space (VIR-TS), texture unit $\vec{T}$, homogeneous equation system, multiclass classifier, digital image recognition

## Abstract

This paper proposes the transformation $S\to \vec{C}$, where **S** is a digital gray-level image and $\vec{C}$ is a vector expressed through the textural space. The proposed transformation is denominated Vectorial Image Representation on the Texture Space (VIR-TS), given that the digital image **S** is represented by the textural vector $\vec{C}$. This vector $\vec{C}$ contains all of the local texture characteristics in the image of interest, and the texture unit $\vec{T}$ entertains a vectorial character, since it is defined through the resolution of a homogeneous equation system. For the application of this transformation, a new classifier for multiple classes is proposed in the texture space, where the vector $\vec{C}$ is employed as a characteristics vector. To verify its efficiency, it was experimentally deployed for the recognition of digital images of tree barks, obtaining an effective performance. In these experiments, the parametric value λ employed to solve the homogeneous equation system does not affect the results of the image classification. The VIR-TS transform possesses potential applications in specific tasks, such as locating missing persons, and the analysis and classification of diagnostic and medical images.

## 1. Introduction

Visual texture is an important element for component classification in scenes and is commonly used for the processing of visual information. The surfaces of all materials are characterized through their texture properties, which can be described as follows: (a) the visual texture is a spatial distribution of gray levels; (b) the visual texture can be perceived through different scales or resolutions; (c) the texture is an area property and not a point property; (d) a region is perceived as texture when the number of primitive objects within it is large. On the other hand, according to reference [1], some important perceptions in the quality of a texture are uniformity, density, rugosity, linearity, direction, frequency, and phase. Henceforth, a texture can be considered as fine, rough, soft, regular, irregular, or linear. The grade of irregularity or the properties of a texture can be found scattered throughout the entire image. In the field of texture analysis, there exist three major problems: (a) texture classification, focused on determining to which class the sampled texture belongs [2,3,4]; (b) texture segmentation, where an image is sectioned into multiple regions and each region has a specific type of texture [5,6]; and (c) texture synthesis, which focuses on constructing a model that can be employed to produce artificial textures for specific applications such as computer graphics [7,8]. Furthermore, according to reference [9], the characteristics extraction techniques can be classified into three categories: geometrical methods, signal processing, and statistical models. Geometrical methods are based on the analysis of primitive textures. Some geometrical methods for primitive extractions include adaptative region extractions, mathematical morphology, structural methods, and border detection [10,11]. The model-based methods hypothesize the subjacent texture, constructing a parametric model that can generate the intensity’s distribution of interest. Ergo, these models can also be employed for texture synthesis. Some of these models that are applied for texture synthesis are called stochastic spatial interaction models, random field models, and fractals [12,13]. The signal processing methods perform an analysis of the frequency components of the images; the latter are also known as filtering methods, and to mention only some of these, we submit spatial domain filter, frequency analysis, and spatial/spatial–frequency methods [14,15]. Last but not least, the statistical methods offer an analysis of the spatial distribution of the local texture characteristics. Such characteristics are represented through a histogram of a variable dimension depending on the procedure employed to calculate the texture unit [16,17,18]. This histogram presents the occurrence frequency of the estimated texture units within the digital image, and its dimension is dependent on the unit texture definition. Selection of the texture extraction method is conducted in agreement with the problem under consideration. There are two types of classifiers for image classification in an a priori knowledge scheme: one-class and multiclass. For one-class classifiers [19,20], an unequivocal class is clearly defined, while the remaining classes are of no interest. In this situation, a region is defined within the characteristics space; this region represents the textural characteristics of the known class. This region is the acceptance zone for the class of interest or is employed as a prototype. On the other hand, in the multiclass classifiers [21,22,23], the characteristics space is divided into multiple regions, each region corresponding to the characteristics of a class and, frequently, the class (image) is represented by a characteristics vector known as a prototype vector. The classification of multiclass images consists of comparing the characteristics vector of a test image with the characteristics vectors of the known classes. Henceforth, the test image is assigned to the class with the most similar characters. This discrimination is performed by means of finding the distance between the vectors within the characteristics space.

To our knowledge, the texture unit has not been defined through a homogeneous equation system, which is defined through an observation window. In this paper, the local texture characteristics are extracted from grayscale images S. To extract the texture characteristics, a mobile observation window of W=3×3 in size is employed to detect local random patterns of P pixels across the image. In each detected position, the pixel values are considered constants within a homogeneous equation system whose solution is the vectorial unit texture $\vec{T}$. This unit $\vec{T}$ is represented in a new texture space as a vector radius that extends from the origin to the vector position $\vec{T}$, such that each random pattern of P pixels has a corresponding texture unit vector $\vec{T}$ (vector radius). By adding together all of the components of the vector radius, $\vec{C}$ is calculated; this latter vector contains all of the local texture characteristics of the image under study, **S**. Ergo, the transformation represents a gray-level image S through a vector $\vec{C}$, whose direction and magnitude depend entirely on the textures of the image. This transformation has been denominated Vectorial Image Representation on the Texture Space (VIR-TS), due to the representation of a digital image **S** through the vector $\vec{C}$. The efficiency of the VIR-TS transform was experimentally corroborated through the classification of tree stem images with a multiclass classifier, where the $\vec{C}$ vector is employed as a characteristics vector.

The report has the following structure. Materials and methods are presented in Section 2. In Section 2.1, the texture space is described based on three subsections: Section 2.1.1, the definition of the texture unit is shown; Section 2.1.2, the definition of the texture unit is represented graphically; and Section 2.1.3, the representation of a digital image in texture space is described. In Section 2.2, the procedure to measure the similarity in texture space between a prototype vector and a test vector is explained. Section 2.3 describes a classifier for multiple classes in texture space and where the VIR-TS vector is used as a feature vector. In Section 3, the experimental work is developed. In Section 3.1, a digital image database is vector-represented in texture space where each vector has its own direction and magnitude. Furthermore, using the vectors obtained in the transformation, the similarity between images is measured. In Section 3.2, experimental results of image classification are reported, which demonstrate the high efficiency of the VIR-TS technique. A discussion of our work is provided in Section 4. Finally, in Section 5 the most relevant conclusions are presented.

## 2. Materials and Methods

### 2.1. Texture Space

#### 2.1.1. Texture Unit Definition

In the texture analysis, a mobile observation window W frequently bears a W=I×J=3×3 size [21,24,25]; it is deployed to extract the local texture characteristics of an image under study. This window is shifted pixel-by-pixel across the whole image and, for each position, the window detects a discrete pattern, which is employed to generate a decimal code called a texture unit. Afterward, the texture unit is interpreted as a discrete variable and is then taken as an index to generate a discrete histogram $h\left(k\right)$. Such a histogram $h\left(k\right)$ is interpreted as a texture spectrum and is then deployed as a characteristics vector in image classifiers [21].

**Figure 1:**
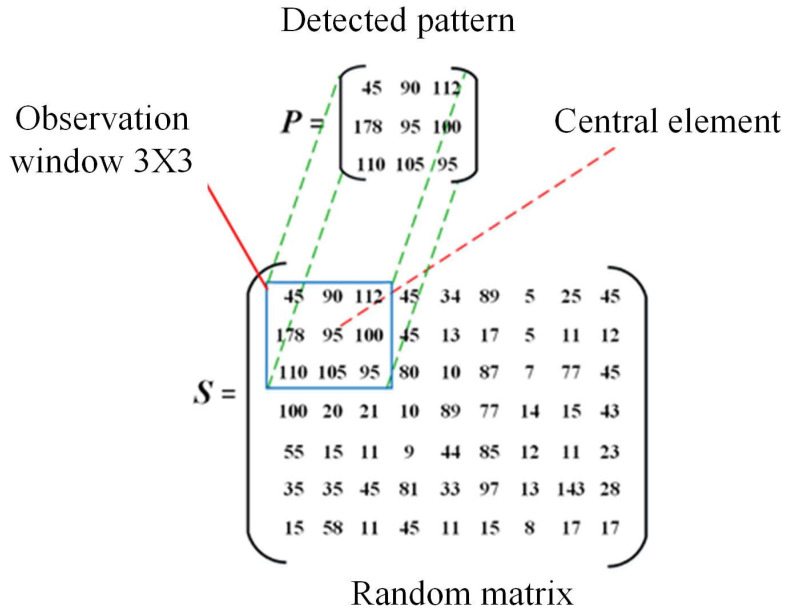
A pattern ***P*** detected in the grayscale image ***S*** through an observation window of 3 × 3 elements.

Now, bearing in mind the structure of the mobile observation window, and considering the gray-level image such as a random matrix $=\left\{s_{m,n}\right\}$ $\left(m=1,2,\ldots ,M;n=1,2,\ldots ,N\right)$, with size M×N, and for each position, a discrete pattern $P=\left\{p_{i,j}\right\}\;\left(I=1,2,3;J=1,2,3\right)$ is detected through the window, as shown in Figure 1. If the pattern elements are considered the coefficient of a homogeneous equation system, the system will be:

(1) $$C_{P}T=0\;\;⟹\;\;\left[\begin{array}{ccc} p_{11} & p_{12} & p_{13}\\ p_{21} & p_{22} & p_{23}\\ \;p_{31} & p_{32} & p_{33} \end{array}\right]\left(\begin{array}{c} t_{1}\\ t_{2}\\ t_{3} \end{array}\right)=\left[\begin{array}{c} 0\\ 0\\ 0 \end{array}\right]\;\;⟹\;\;\begin{array}{c} p_{11}t_{1}+p_{12}t_{2}+p_{13}t_{3}=0\\ p_{21}t_{1}+p_{22}t_{2}+p_{23}t_{3}=0\\ p_{31}t_{1}+p_{32}t_{2}+p_{33}t_{3}=0 \end{array}$$

where $C_{P=}\left[\begin{array}{ccc} p_{11} & p_{12} & p_{13}\\ p_{21} & p_{22} & p_{23}\\ \;p_{31} & p_{32} & p_{33} \end{array}\right]$ is termed the coefficient matrix of the homogeneous linear system, represented as a matrix of 3×3 real elements, and $T=\left[\begin{array}{c} t_{1}\\ t_{2}\\ t_{3} \end{array}\right]$ is called the unit texture vector. The trivial solution of the homogeneous equation system occurs when all of the elements of vector T have a value of zero: $t_{1}=0$, $t_{2}=0$, $t_{3}=0$. Nonetheless, this solution is not functional for our interests; thus, a nontrivial solution must be found. Therefore, based on a linear algebra concept, the nontrivial solution is possible when its determinant is equal to zero; as a consequence, there will be infinite solutions. To achieve this, the term K is introduced within the equations and their determinant is equal to zero, as shown in Equation (2):

(2) $$det\left|C_{p}\right|=\left|\begin{array}{ccc} p_{11} & p_{12} & p_{13}\\ p_{21} & p_{22} & p_{23}\\ \;p_{31} & p_{32} & Kp_{33} \end{array}\right|=0$$

Hence, the problem becomes that in finding a *K* value, so that the condition $det\left|C_{p}\right|=0$ is satisfied. From Equation (2), in terms of the matrix elements $C_{P}$, *K* has a value of

(3) $$K=\frac{p_{31}\left(p_{12}p_{23}-p_{22}p_{13}\right)+p_{32}\left(p_{13}p_{21}-p_{23}p_{11}\right)}{p_{33}\left(p_{21}p_{12}-p_{11}p_{22}\right)}$$

Once the value K is determined, it is introduced into the equation system; Equation (1) then takes the following form:

(4) $$\begin{array}{c} p_{11}t_{1}+p_{12}t_{2}+p_{13}t_{3}=0\\ p_{21}t_{1}+p_{22}t_{2}+p_{23}t_{3}=0\\ p_{31}t_{1}+p_{32}t_{2}+Kp_{33}t_{3}=0 \end{array}$$

where the value K is determined by Equation (3).

Afterward, to determine the texture unit T, the nontrivial solution of Equation (4) must be found. As a first step, the first two linear equations are left depending on $t_{3}$:

(5) $$\begin{array}{c} p_{11}t_{1}+p_{12}t_{2}=p_{13}t_{3}\\ p_{21}t_{1}+p_{22}t_{2}=p_{23}t_{3} \end{array}\;$$

employing the Cramer Rule method, the solution for $t_{1}$ is obtained through:

(6) $$t_{1}=\frac{Dt_{1}}{D}=\frac{\left|\begin{array}{cc} p_{13}t_{3} & p_{12}\\ p_{23}t_{3} & p_{22} \end{array}\right|}{\left|\begin{array}{cc} p_{11} & p_{12}\\ p_{21} & p_{22} \end{array}\right|}=\frac{p_{13}p_{22}-p_{23}p_{12}}{p_{11}p_{22}-p_{12}p_{21}}t_{3}\;$$

while the solution for $t_{2}$ is:

(7) $$t_{2}=\frac{Dt_{2}}{D}=\frac{\left|\begin{array}{cc} p_{11} & p_{13}t_{3}\\ p_{21} & p_{23}t_{3} \end{array}\right|}{\left|\begin{array}{cc} p_{11} & p_{12}\\ p_{21} & p_{22} \end{array}\right|}=\frac{p_{11}p_{23}-p_{21}p_{13}}{p_{11}p_{22}-p_{12}p_{21}}t_{3}$$

where D is the determinant of the 2 × 2 equation system, $Dt_{1}$ is the determinant for $t_{1}$, and $Dt_{2}$ is the determinant for $t_{2}$. It is noteworthy that $t_{1}$ and $t_{2}$ function on the basis of $t_{3}$; accordingly, the infinite solution in parametric form is:

(8) $$\infty -solutions\left\{\begin{array}{cc} \begin{array}{c} t_{1}=\frac{p_{13}p_{22}-p_{23}p_{12}}{p_{11}p_{22}-p_{12}p_{21}}\lambda \\ t_{2}=\frac{p_{1,1}p_{2,3}-p_{21}p_{13}}{p_{11}p_{22}-p_{12}p_{21}}\lambda \\ t_{3}=\lambda \end{array} & \lambda \in \mathbb{R} \end{array}\right.$$

Observing Expression (8), for each real value of lambda λ, a unique resolution of the infinite solution is found. For example, when λ=0, the trivial solution of the equation system is obtained ($t_{1}=0$, $t_{2}=0$, and $t_{3}=0$); henceforth, the nontrivial solution is obtained when λ≠0.

#### 2.1.2. Graphical Representation

**Figure 2:**
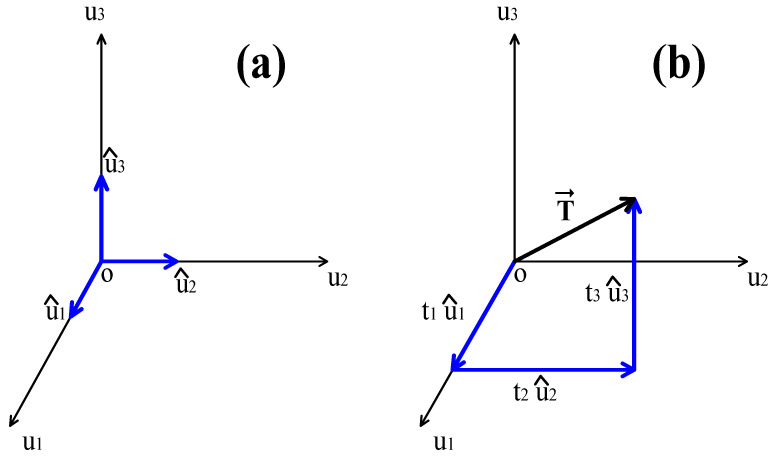
Representation of unit $\vec{T}$ in the texture space: (**a**) graphic representation of unit vectors ${\hat{u}}_{1}$, ${\hat{u}}_{2}$, ${\hat{u}}_{3}$; (**b**) graphic representation of texture unit $\vec{T}$ and its components $t_{1}\;{\hat{u}}_{1}+t_{2}\;{\hat{u}}_{2}+t_{3}\;{\hat{u}}_{3}$.

Based on Equation (1) and Expression (8), the unit texture vector is defined by: $T=\left[\begin{array}{c} t_{1}\\ t_{2}\\ t_{3} \end{array}\right]=\left[\begin{array}{c} \frac{p_{13}p_{22}-p_{23}p_{12}}{p_{11}p_{22}-p_{12}p_{21}}\lambda \\ \frac{p_{11}p_{23}-p_{21}p_{13}}{p_{11}p_{22}-p_{12}p_{21}}\lambda \\ \lambda \end{array}\right].$ It can be represented through the Cartesian coordinate system form:

(9) $$\vec{T}=t_{1}\;{\hat{u}}_{1}+t_{2}\;{\hat{u}}_{2}+t_{3}\;{\hat{u}}_{3}=\frac{p_{13}p_{22}-p_{23}p_{12}}{p_{11}p_{22}-p_{12}p_{21}}\lambda {\hat{u}}_{1}+\frac{p_{11}p_{23}-p_{21}p_{13}}{p_{11}p_{22}-p_{12}p_{21}}\lambda {\hat{u}}_{2}+\lambda {\hat{u}}_{3}$$

where ${\hat{u}}_{1}$, ${\hat{u}}_{2}$, ${\hat{u}}_{3}$ are the unit vectors that indicate the axis direction in a rectangular coordinate system of three dimensions (Figure 2a). Hereafter (9), the $\frac{p_{13}p_{22}-p_{23}p_{12}}{p_{11}p_{22}-p_{12}p_{21}}\lambda ,\frac{p_{11}p_{23}-p_{21}p_{13}}{p_{11}p_{22}-p_{12}p_{21}}\lambda ,\lambda$ scalars are the components of vector $\vec{T}$ in the directions $u_{1},\;u_{2},\;u_{3}$. Finally, from Equation (8), the magnitude of vector $\vec{T}$ is:

(10) $$\left|\vec{T}\right|=\sqrt{{\left(\frac{p_{13}p_{22}-p_{23}p_{12}}{p_{11}p_{22}-p_{12}p_{21}}\lambda \right)}^{2}+{\left(\frac{p_{11}p_{23}-p_{21}p_{13}}{p_{11}p_{22}-p_{12}p_{21}}\lambda \right)}^{2}+\lambda^{2}}$$

and its directing cosines are:

(11) $$\begin{array}{c} cos\alpha =\frac{\frac{p_{13}p_{22}-p_{23}p_{12}}{p_{11}p_{22}-p_{12}p_{21}}\lambda }{\left|\vec{T}\right|}\\ cos\beta =\frac{\frac{p_{11}p_{23}-p_{21}p_{13}}{p_{11}p_{22}-p_{12}p_{21}}\lambda }{\left|\vec{T}\right|}\\ cos\gamma =\frac{\lambda }{\left|\vec{T}\right|} \end{array}.$$

where

(12) $$cos^{2}\alpha +cos^{2}\beta +cos^{2}\gamma =1.$$

with $\left|\vec{T}\right|$ being the magnitude of vector $\vec{T}$; its graphic presentation is displayed in Figure 2b. Based on Figure 2b, the texture unit $\vec{T}$ is a radius vector that extends from the origin to the coordinates $t_{1}=\frac{p_{13}p_{22}-p_{23}p_{12}}{p_{11}p_{22}-p_{12}p_{21}}\lambda ,\;t_{2}=,\frac{p_{11}p_{23}-p_{21}p_{13}}{p_{11}p_{22}-p_{12}p_{21}}\lambda ,t_{3}=\lambda$.

It is clear that the direction and magnitude depend on the λ value and the elements in the P pattern.

#### 2.1.3. Image Representation on the Texture Space

Given that, if a grayscale image S has an M×N size and if this image is analyzed through an I×J window, then there are $\left(M-I+1\right)\times \left(N-J+1\right)$ patterns P. Furthermore, given that each P pattern (in the image domain) generates a texture unit $\vec{T}$ (in texture space), then when the image S is analyzed locally through the observation window for the n−th pattern $P_{n}$ $\left(n=1,2,3,\ldots ,N_{P}=\left(M-I+1\right)\times \left(N-J+1\right)\right)$, the n−th texture unit ${\vec{T}}_{n}$ is calculated (radius vector in texture space); as a consequence, the image S can be represented through a series of radius vectors. Thus, adding together all of the components of all of these radius vectors in the texture space, the image S is represented by vector $\vec{C}$, defined as:

(13) $$\vec{C}=a_{1}{\hat{u}}_{1}+a_{2}{\hat{u}}_{2}+a_{3}{\hat{u}}_{3}$$

**Figure 3:**
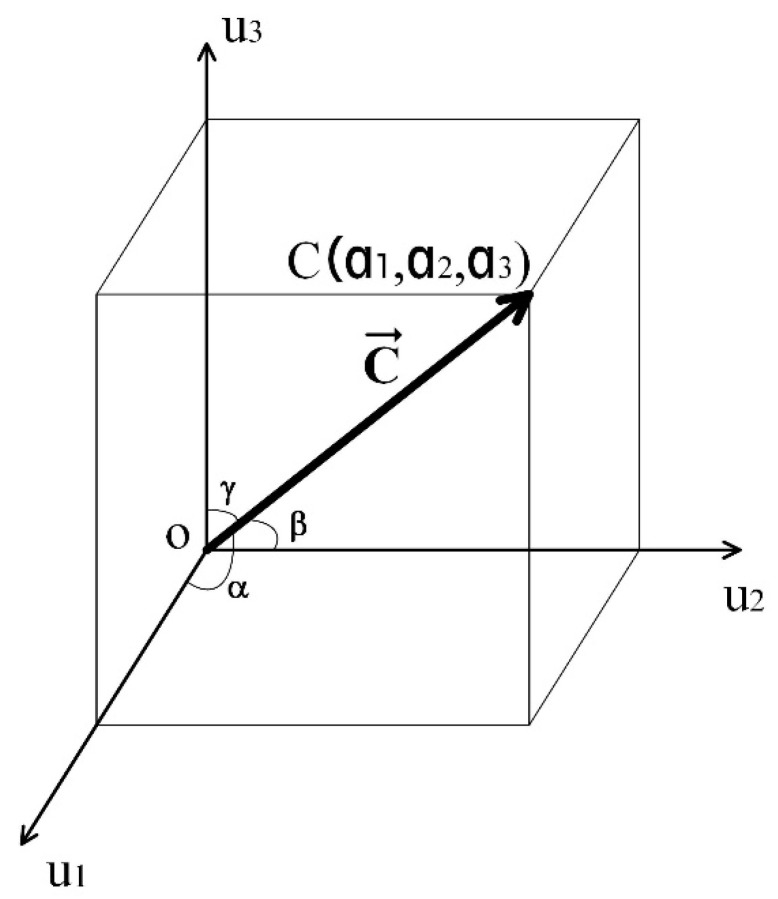
Graphic representation of the texture vector $\vec{C}$ with its directing cosines.

The directions are given by ${\hat{u}}_{1},{\hat{u}}_{2},{\hat{u}}_{3}$ and the components $a_{1},a_{2},a_{3}$ are calculated with:

(14) $$\begin{array}{c} a_{1}=\overset{N_{P}=\left(M-I+1\right)\times \left(N-J+1\right)}{\underset{n=1}{\sum }}t_{1n}\\ a_{2}=\overset{N_{P}=\left(M-I+1\right)\times \left(N-J+1\right)}{\underset{n=1}{\sum }}t_{2n}\\ a_{3}=\overset{N_{P}=\left(M-I+1\right)\times \left(N-J+1\right)}{\underset{n=1}{\sum }}t_{3n} \end{array},$$

where $t_{1n}$ is the n−th component of the elements for $t_{1}$, $t_{2n}$ is the n−th component of the elements for $t_{2}$, and $t_{3n}$ is the n−th component of the elements for $t_{3}$. Equation (9) was considered, and $N_{P}$ is the total of the patterns found in the digital image under study. Figure 3 depicts vector $\vec{C}$, which is in texture space.

Considering Figure 3 and Equation (13), the magnitude of vector $\vec{C}$ is:

(15) $$\left|\vec{C}\right|=\sqrt{a_{1}^{2}+a_{2}^{2}+a_{3}^{2}}$$

where its directing cosines are given with:

(16) $$\begin{array}{c} cos\alpha =\frac{a_{1}}{\left|\vec{C}\right|}\\ cos\beta =\frac{a_{2}}{\left|\vec{C}\right|}\\ cos\gamma =\frac{a_{3}}{\left|\vec{C}\right|} \end{array}$$

and holding the equivalence:

(17) $$cos^{2}\left(\frac{a_{1}}{\left|\vec{C}\right|}\right)+cos^{2}\left(\frac{a_{2}}{\left|\vec{C}\right|}\right)+cos^{2}\left(\frac{a_{3}}{\left|\vec{C}\right|}\right)=1$$

Based on the performed analysis, image S can be represented as a radius vector $\left|\vec{C}\right|$ in the texture space whose magnitude and direction depend on the randomness in the image under study.

### 2.2. Similarity Measurement between a Prototype Image and Test Image

With the knowledge that the $S\to \vec{C}$ transformation is possible, then the measurement of similarity between a prototype image and an unknown test image can be performed in the texture space.

Given a digital image $S_{c}$ of a c class whose texture vector is ${\vec{C}}_{c}$, and given an unknown test image $S_{Test}$ whose vector is ${\vec{C}}_{Test}$, the difference between the $S_{c}$ and $S_{Test}$ images in the texture space can be calculated through subtraction of the unknown image ${\vec{C}}_{Test}$ minus the vector of the prototype image ${\vec{C}}_{c}$:

(18) $${\vec{C}}_{dif}={\vec{C}}_{Test}-{\vec{C}}_{c}$$

where ${\vec{C}}_{dif}$ is the difference vector between the texture images.

**Figure 4:**
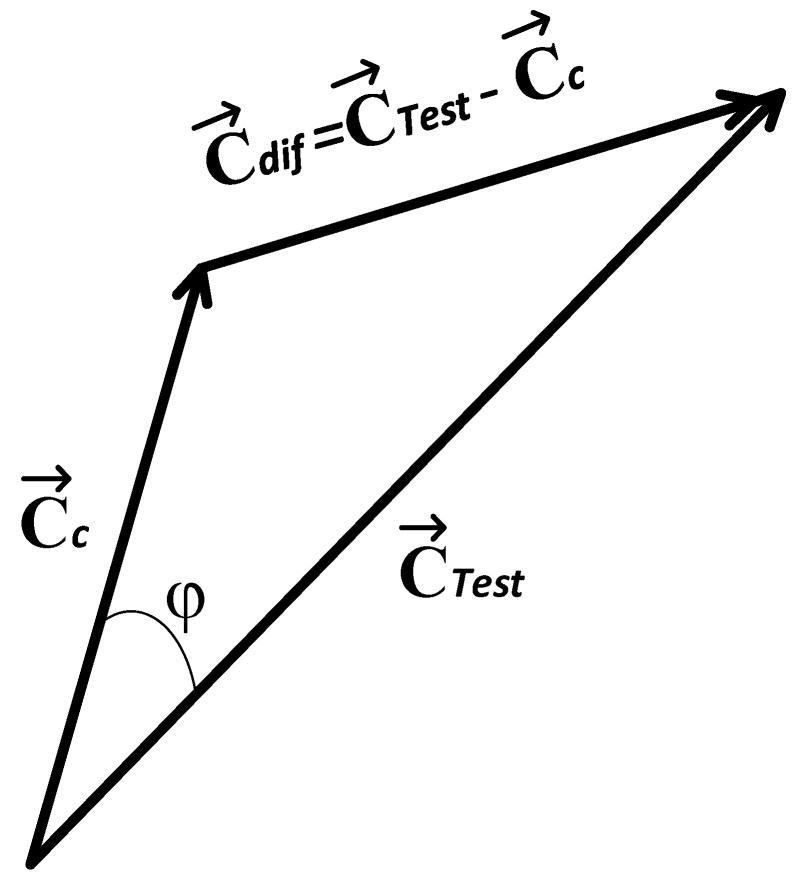
Geometry employed for the similarity measurement between $S_{c}$ and $S_{Test}$.

Images deploy the ${\vec{C}}_{c}$ and ${\vec{C}}_{Test}$ vectors. Considering the cosines law and the geometry present in Figure 4, we obtain:

(19) $${\left|{\vec{C}}_{dif}\right|}^{2}={\left|{\vec{C}}_{Test}\right|}^{2}+{\left|{\vec{C}}_{c}\right|}^{2}-2\left|{\vec{C}}_{Test}\right|\left|{\vec{C}}_{c}\right|cos\varphi$$

and from (19), we obtain:

(20) $$2\left|{\vec{C}}_{Test}\right|\left|{\vec{C}}_{c}\right|cos\varphi ={\left|{\vec{C}}_{Test}\right|}^{2}+{\left|{\vec{C}}_{c}\right|}^{2}-{\left|{\vec{C}}_{dif}\right|}^{2}$$

Due to the geometry of the problem, if (18) is substituted in (20), we obtain:

(21) $$2\left|{\vec{C}}_{Test}\right|\left|{\vec{C}}_{c}\right|cos\varphi ={\left|{\vec{C}}_{Test}\right|}^{2}+{\left|{\vec{C}}_{c}\right|}^{2}-{\left|{\vec{C}}_{Test}-{\vec{C}}_{c}\right|}^{2}$$

On applying the distributive law:

(22) $$2\left|{\vec{C}}_{Test}\right|\left|{\vec{C}}_{c}\right|cos\varphi ={\vec{C}}_{Test}\cdot {\vec{C}}_{Test}+{\vec{C}}_{c}\cdot {\vec{C}}_{c}-{\vec{C}}_{Test}\cdot {\vec{C}}_{Test}+{\vec{C}}_{Test}\cdot {\vec{C}}_{c}-{\vec{C}}_{c}\cdot {\vec{C}}_{c}+{\vec{C}}_{c}\cdot {\vec{C}}_{Test}$$

On reducing, we reach

(23) $$2\left|{\vec{C}}_{Test}\right|\left|{\vec{C}}_{c}\right|cos\varphi =2\left({\vec{C}}_{Test}\cdot {\vec{C}}_{c}\right)$$

**Figure 5:**
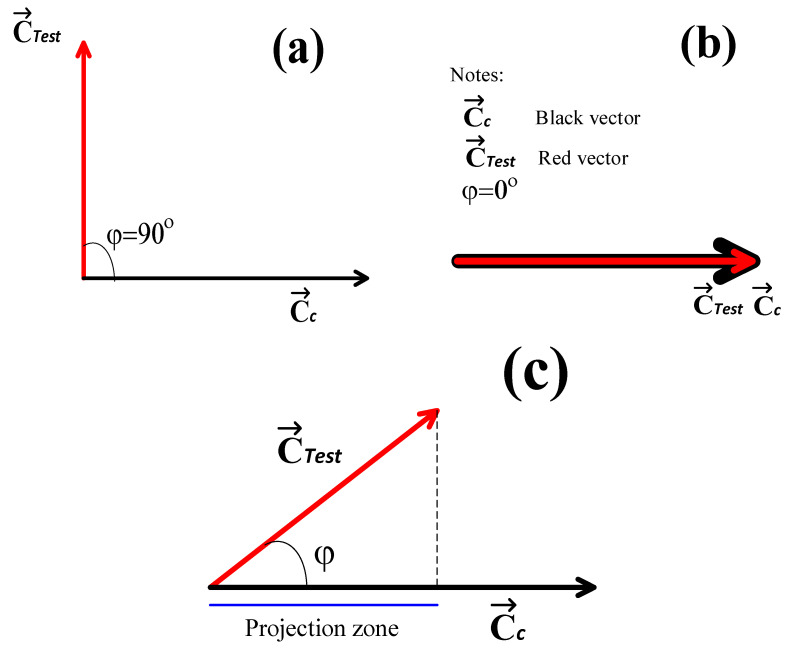
(**a**) The ${\vec{C}}_{Test}$ and the ${\vec{C}}_{c}$ vectors are orthogonal, and the similarity of $S_{Test}$ and $S_{c}$ equals 0. (**b**) The ${\vec{C}}_{Test}$ and ${\vec{C}}_{c}$ vectors are parallel; hence, the $S_{Test}$ and $S_{c}$ images are identical, and (**c**) there is a certain angle between the ${\vec{C}}_{Test}$ and the ${\vec{C}}_{c}$ vectors; thus, the $S_{Test}$ and $S_{c}$ images possess a certain degree of similarity.

From (23), the following relationship can be achieved:

(24) $$cos\varphi =\frac{{\vec{C}}_{Test}\cdot {\vec{C}}_{c}}{\left|{\vec{C}}_{Test}\right|\left|{\vec{C}}_{c}\right|}$$

where the symbol indicates a scalar product, $\left|{\vec{C}}_{Test}\right|$ is the magnitude of vector ${\vec{C}}_{Test}$, $\left|{\vec{C}}_{c}\right|$ is the magnitude of vector ${\vec{C}}_{c}$, and cosφ is the cosine of the angle formed between the ${\vec{C}}_{Test}$ and ${\vec{C}}_{c}$ vectors. With the knowledge that Expression (24) is employed to measure the similarity between vectors, this equivalence is achieved:

(25) $$sim\left(S_{Test},S_{c}\right)=cos\varphi =\frac{{\vec{C}}_{Test}\cdot {\vec{C}}_{c}}{\left|{\vec{C}}_{Test}\right|\left|{\vec{C}}_{c}\right|}$$

where sim(S_Test, S_c) is the similarity measurement between the $S_{Test}$ and $S_{c}$ images. Thus, based on Figure 4 and Equation (25), the following conditions (as points) can be indicated:

1. If cosφ=0, then $sim\left(S_{Test},S_{c}\right)=0$, because ${\vec{C}}_{Test}$ and ${\vec{C}}_{c}$ are orthogonal, φ=90°. Ergo, the $S_{Test}$ and $S_{c}$ images are completely different (see Figure 5a).
2. If cosφ=1, then $sim\left(S_{Test},S_{c}\right)=1$, because ${\vec{C}}_{Test}$ and ${\vec{C}}_{c}$ have the same direction and magnitude, φ=0°. For this case, the $S_{Test}$ and $S_{c}$ images are identical (see Figure 5b).
3. If 0<cosφ<1, then $0<sim\left(S_{Test},S_{c}\right)<1$; consequently, the $S_{Test}$ and $S_{c}$ images have a certain degree of similarity between them, given that the ${\vec{C}}_{Test}$ and ${\vec{C}}_{c}$ vectors are not parallel within the texture space. Therefore, the condition 0°<φ<90° is satisfied (see Figure 5c).

Based on conditions 1–3 and on Figure 5, it is possible to measure the similarity between images within the texture space; therefore, texture image classification is also a possibility.

### 2.3. Image Classification in the Texture Space

**Figure 6:**
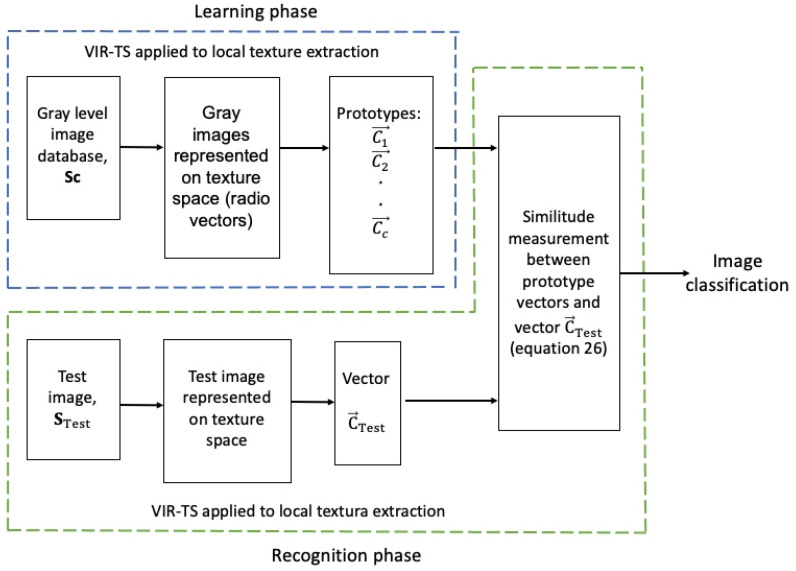
A schematic representation of the multiclass classifier.

Figure 6 schematically displays the proposed multiclass classifier for image recognition within the texture space. The classifier consists of two phases: learning and recognition. During the learning phase, a human expert identifies and names a known image database $S_{c}\;\left(c=1,2,3,\ldots ,C\right)$, where each image is considered as an independent class; each class has a series of radius vectors ${\vec{T}}_{n}$ that are calculated, and with these radius vectors, the prototype vector ${\vec{C}}_{c}$ is obtained. This ${\vec{C}}_{c}$ vector represents all of the local texture characteristics of the image $S_{c}$ within the c class. In the recognition phase, an unknown test image $S_{Test}$ is represented through a series of radius vectors ${\vec{T}}_{t}\left(t=n\right),$ and the ${\vec{C}}_{Test}$ vector is calculated with these. Afterward, the similarity between the test image $S_{Test}$ and the prototype images $S_{c}$ is measured in the texture space employing Expression (25). The test image $S_{Test}$ is then assigned to the most similar class; such a condition is achieved when the angle y is the smallest of these during the comparison between the ${\vec{C}}_{Test}$ and ${\vec{C}}_{c}$ vectors (see Figure 5) and when the following condition is satisfied:

(26) $$max\left(sim\left(S_{Test},S_{c}\right)\right)=max\left(cos\varphi \right)=\frac{{\vec{C}}_{Test}\cdot {\vec{C}}_{c}}{\left|{\vec{C}}_{Test}\right|\left|{\vec{C}}_{c}\right|}$$

Ergo, the image $S_{Test}$ is assigned to the c class when the projection of the vector ${\vec{C}}_{Test}$ above the ${\vec{C}}_{c}$ vector is the unit or that closest to the unit.

The classifier results are displayed in a confusion matrix $H=\left\{h_{cc}\right\}$; the rows show the prototype images, the columns show the test images, the elements of the main diagonal correspond to the correct classification hits, and the elements outside of the diagonal represent the classification errors. The classification efficiency in terms of percentage is calculated with:

(27) $$Ef\%=\frac{\sum diag\left(\left\{h_{cc}\right\}\right)}{\sum_{c}\sum_{c}h_{cc}}\times 100$$

where Ef% is the efficiency in terms of percentage, $\sum diag\left(\left\{h_{cc}\right\}\right)$ is the sum of all of the elements of the main diagonal in the confusion matrix, and $\underset{c}{\sum }\underset{c}{\sum }h_{cc}$ is the sum of all of the elements within the confusion matrix.

## 3. Experimental Work and Results

### 3.1. Transformation of an Image S Onto a Texture Vector $\vec{C}$

**Figure 7:**
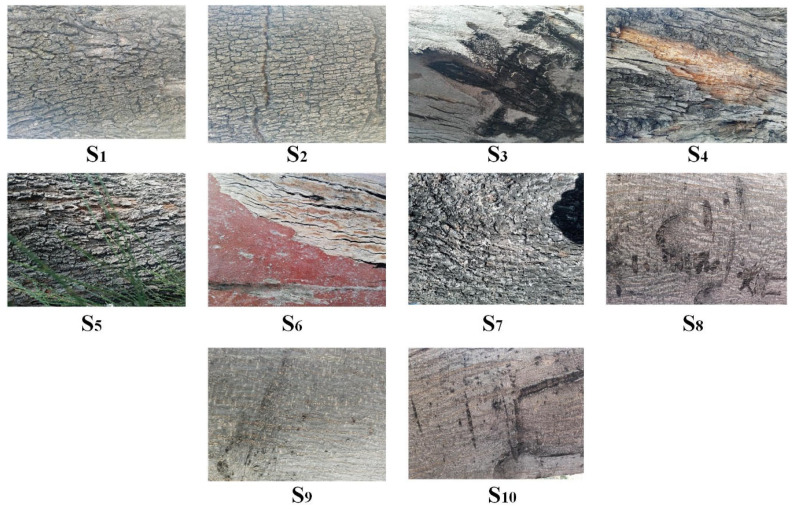
Digital images of the tree stems employed in the experiments.

In this section, a database comprising 10 digital images of tree stems $S_{c}\left(c=1,2,\ldots ,10\right)$ is represented through texture vectors ${\vec{C}}_{c}$, employing λ=2 and λ=25 values and an observation window of W=3×3 size. The database is presented in Figure 7. Each image $S_{c}$ was acquired with a Smartphone LG 50, and rotation and scale are controlled under natural illumination and with a fixed resolution of M×N=3120×4160 pixels.

Additionally, the $S_{c}\to {\vec{C}}_{c}$ transformation was performed applying the following steps: (a) the RGB image acquired with the Smartphone LG 50 was transformed into a grayscale level $S_{c}$ deploying MatLab 2016b^®^ scientific software; (b) an observation window with a W=3×3 size is selected; (c) the window W is displaced element-by- element across the entire gray-level image $S_{c}$ with a M×N=3120×4160 size; (d) for each pattern P, a homogeneous equation system is proposed, then its $\vec{T}$ unit is calculated; (e) all units $\vec{T}$ are represented in the texture space as a radius vector, and (f) by adding together all of the radius vectors, the vector ${\vec{C}}_{c}$ is estimated. Exercising steps a–f, the images in Figure 7 were represented through a texture vector ${\vec{C}}_{c}\left(c=1,2,3,\ldots ,10\right)$. The results are presented in Table 1.

Considering Figure 7 and Table 1, the digital image $S_{c}$ is represented in the texture space through a radius vector ${\vec{C}}_{c}$, whose components are dependent on the texture characteristics of the image and on the parametrization value λ. During the transformation, the texture characteristics of the image render the ${\vec{C}}_{c}$ vector unique in the texture space, while the parameter λ operates as a scale factor.

To verify the uniqueness of each vector in Table 1, the similarity between these is measured employing the scalar product in Equation (25). The results are displayed in a confusion matrix, where the elements of the main diagonal correspond to the similarity measurements of the same vector $\left({\vec{C}}_{c}\;y\;{\vec{C}}_{c}\right);$ hence, its value is the unit (marked in blue). Otherwise, the elements outside of the main diagonal correspond to the similarity measurement between two different vectors $\left({\vec{C}}_{c}\;y\;{\vec{C}}_{m}\right)$; consequently, such elements have a value lower than the unit. Table 2 and Table 3 present these results: 

Based on the results of Table 2 and Table 3, both confusion matrixes are identical, given that the elements in their respective diagonals are the unit, and the elements outside of their diagonals are fewer than the unit. This corroborates that a digital image $S_{c}$ is represented in the texture space through a unique vector ${\vec{C}}_{c}$, and that the parameter λ, operated as a scale factor and its value, does not affect the results. Furthermore, the similarity measurements between images above 0.94 are attributed to the parametrization of the homogeneous equation system due to its resolution. This causes the third component of all of the vectors to bear the same value λ, and the remaining two components (first and second) are the only components scaled by the value of λ (see Equation (8)).

### 3.2. Image Recognition in the Texture Space

Knowing that each digital image can be represented in the texture space through a vector, the goal of this section is to prove that the digital images can be classified in the texture space. As previously presented in Figure 7, the database consists of 10 digital images with a size of M×N=3120×4160 pixels; these images show the bark of tree stems and were acquired under natural lighting and controlled scale and rotation. The classifier employed for image recognition was described in Section 4. In both phases, the same images are employed for both learning and recognition, along with the same observation window size of W=3×3 pixels. The similarity measurement in the texture space is performed considering the maximal likeness between the ${\vec{C}}_{Test}$ and ${\vec{C}}_{c}$ vectors (Equations (25) and (26)). To conclude, the classification results are presented through two confusion matrixes: Table 4 displays the confusion matrix for λ=2, and Table 5 presents the confusion matrix for λ=25.

It is worth recalling that the elements of the main diagonal in these matrixes represent the correct classification hits, and the elements outside of the main diagonal are the identification errors. In this manner, based on Equation (27) and Table 4 and Table 5, the classification is:

(28) $$\begin{array}{c} Ef_{\lambda =2}\%=\frac{1+1+1+1+1+1+1+1+1+1}{10}=100\%\;\;\;\;\left(\mathrm{Table}\;4\right)\\ Ef_{\lambda =25}\%=\frac{1+1+1+1+1+1+1+1+1+1}{10}=100\%\;\;\;\;\left(\mathrm{Table}\;5\right) \end{array}$$

where $Ef_{\lambda =2}\%$ is the image classification efficiency in terms of percentage for λ=2, and $Ef_{\lambda =25}\%$ is the image classification efficiency for λ=25. The efficiency is 100% in both cases. This further confirms that the proposed transformation in Section 2, along with the classifier described in Section 4, entertain a high efficiency and that the recognition of the images can be performed in the texture space. The high efficiency is attributed to the following points:

1. In the $S\to \vec{C}$ transformation, image S is completely characterized through its local texture characteristics, and these are represented by the texture vector $\vec{C}$.
2. The digital image is essentially a field of randomness, given the nature of the light source and the noise detected by the system; henceforth, for each image $S_{c}$, a unique vector ${\vec{C}}_{c}$ is generated in the texture space with a particular direction and magnitude that differ for each class.

Nonetheless, the efficiency of our proposal can be reduced if the digital images are classified dynamically (in real time). This is due to the fluctuation in the light source temporarily and spatially. Consequently, for each instant of time, the pixels of the digital camera vary in intensity. In other words, the noise during the acquisition of the image increases; thus, the texture vector $\vec{C}$ changes, causing recognition errors.

## 4. Discussion

In this paper, the $S\to \vec{C}$ transformation is proposed where S is a grayscale image and $\vec{C}$ is a vector in a new space, which is denominated texture space. Essentially, the transformation consists of representing the image S through a series of radius vectors in the texture space, with each radius vector a texture unit $\vec{T}$, and this is calculated by solving a homogeneous equation system. Afterward, the vector $\vec{C}$ is calculated by the sum of all of the radius vectors and, subsequently, all of the local texture characteristics of the image under study are considered in it. Its direction and magnitude are in agreement with the randomness in the digital image and, for each image $S_{c}$, a unique vector ${\vec{C}}_{c}$ is generated. Additionally, a multiclass classifier is proposed and applied within the texture space where the vector ${\vec{C}}_{c}$ is employed as a characteristics vector, demonstrating its potential application for image classification. Based on these results, the following points are worth mentioning:

1. The image S is fully characterized in the transformation $\left(S\to \vec{C}\right)$, where the texture space is represented by the texture vector $\vec{C}$. The new transformation can be termed Vectorial Image Representation on the Texture Space (VIR-TS) because, in the image transformation, the image S comes to be represented by the vector $\vec{C}$.
2. Due to the irregular nature of the light source and the noise during the photodetection process, the image S is considered a field of randomness; consequently, a unique vector $\vec{C}$ is generated for each digital image (see Table 1).
3. The vector $\vec{C}$ withholds all local texture characteristics of the image under study, given that the vector is calculated by the sum of all of the radius vectors, where a radius vector is defined as texture unit $\vec{T}$.
4. The texture unit $\vec{T}$ possesses a vectorial character because it is calculated by solving a homogeneous equation system of 3×3.
5. The texture vector $\vec{C}$ can be employed as a characteristics vector in classifiers with a high efficiency (see Table 3 and Table 4).
6. The value λ employed for the solution of the homogeneous equation system does not affect the results of the image recognition.
7. The $S\to \vec{C}$ transformation has a potential application in the development of artificial vision systems focused on the recognition of digital images.
8. In the experimental work, the number of classes does not affect the results of the classification efficiency, given that each digital image is represented by its own vector $\vec{C}$ in the texture space (see point 2).
9. Because medical images contain local textural features that can be extracted through local analysis [3,4,26,27], and knowing that the technique reported in this work also extracts texture features based on local analysis, then the VIR-TS transform and the classifier described in Section 2.3 can be applied in medical image recognition. The benefit would be the development of medical diagnostic systems with high efficiency, easy to implement because the definition of the texture unit is based on a linear transformation and not on pattern encoding [21,28], where the overflow of physical memory of the computer is possible [29].
10. Comparing the statistical texture extraction techniques reported in reference [21] with the VIR-TS technique based on linear transformations, both texture extraction techniques are very different. In statistical techniques, the texture unit is calculated based on the encoding of discrete random patterns located on the digital image, its texture unit is considered a random event and the texture characteristics are represented through a discrete histogram. In our technique called VIR-TS, the texture unit is calculated based on a linear transformation, its texture unit is a radius vector, and the texture features are represented in a texture space through a random vector.

The Vector Image Representation on the Texture Space (VIR-TS) transformation is very different from the statistical techniques reported in reference [21]. In the VIR-TS transformation, the texture unit is a radius vector, the vector is calculated by solving a homogeneous system of equations, and its graph can be visualized in the texture space. With the transformation, the digital image *S* is expressed in the texture space by the random vector $\vec{C}$, which consists of three components, $a_{1},a_{2},a_{3}$, and whose addresses are ${\hat{u}}_{1},{\hat{u}}_{2},{\hat{u}}_{3}$. Because the image is vector-represented, image classification in texture space is performed by measuring the similarity between the prototype vectors and the test vector. Their similarity is calculated through the projection between both vectors. Finally, the test image is assigned to the most similar class. Based on the experimental work, the VIR-TS transformation has high classification efficiency because its texture feature extraction efficiency is very high. Furthermore, its implementation is very easy because the digital image is represented through a three-component random vector.

With the knowledge that our proposal has potential application in image recognition, our future lines of research will include rendering the VIR-TS transform invariant to rotation and scale; proposing the VIR-TS transform for color image classification; applying the VIR-TS transform in the recognition of biomedical images; and performing an efficiency study of classification in images with noise.

## 5. Conclusions

In this paper, the Vectorial Image Representation on the Texture Space (VIR-TS) transform is proposed and applied. The VIR-TS transform is based on the extraction of local texture characters in the image S and represents these through the vector $\vec{C}$ in the texture space. Each radius vector is a texture unit $\vec{T}$, which is estimated by solving a homogeneous equation system of 3 × 3. In the texture space, each image has a corresponding unique vector, given that the image is a random field of pixels. Experimentally, the vector $\vec{C}$ was employed as a characteristics vector in a new multiclass classifier; thus, the high efficiency of the VIR-TS transform was corroborated through the classification of tree stem digital images. The efficiency reached 100%; however, in applications under natural environments, its efficiency may be significantly less due to the noise in photodetections and the random nature of light.

The VIR-TS transform has potential application in locating missing persons and classifying medical images.

## Figures and Tables

**Table 1:** Texture vectors ${\vec{C}}_{c}$ obtained from the digital images shown in Figure 6.

$\mathrm{Transformation}\;\mathrm{from}\;\mathrm{Image}\;\mathrm{to}\;S_{c}\to {\vec{C}}_{c}$ Vector	$\mathrm{Vectors}\;\vec{C}$ Obtained for λ=2	$\mathrm{Vectors}\;\vec{C}\;$ Obtained for λ=25
$S_{1}\to {\vec{C}}_{1}$	$9.56\times 10^{2}\;{\hat{u}}_{1}$ + 1.65×10^5^${\hat{u}}_{2}$ + 177608 ${\hat{u}}_{3}$	$11.9\times 10^{5\;}{\hat{u}}_{1}+2\times 10^{5\;}{\hat{u}}_{2}+22.2\times 10^{5\;}{\hat{u}}_{3}$
$S_{2}\to {\vec{C}}_{2}$	−2.38×10^4^${\hat{u}}_{1}$ + 1.97×10^5^ ${\hat{u}}_{2}$ + 177608 ${\hat{u}}_{3}$	$-2.98\times 10^{5}{\hat{u}}_{1}+2.465\times 10^{6\;}{\hat{u}}_{2}+22.2\times 10^{5\;}{\hat{u}}_{3}$
$S_{3}\to {\vec{C}}_{3}$	$-8.73\times 10^{4\;\;}{\hat{u}}_{1}$ + 2.09×10^5^ ${\hat{u}}_{2}$ + 177608 ${\hat{u}}_{3}$	$-10.91\times 10^{5\;\;}{\hat{u}}_{1}+2.621\times 10^{6\;}{\hat{u}}_{2}+22.2\times 10^{5\;}{\hat{u}}_{3}$
$S_{4}\to {\vec{C}}_{4}$	$4.44\times 10^{3\;\;}{\hat{u}}_{1}$ + 1.70×10^5^ ${\hat{u}}_{2}$ + 177608 ${\hat{u}}_{3}$	$5.55\times 10^{4\;\;}{\hat{u}}_{1}+2.136\times 10^{6\;}{\hat{u}}_{2}+22.2\times 10^{5\;}{\hat{u}}_{3}$
$S_{5}\to {\vec{C}}_{5}$	$-6.06\times 10^{3\;\;}{\hat{u}}_{1}$ + 1.81×10^5^ ${\hat{u}}_{2}$ + 177608 ${\hat{u}}_{3}$	$-7.58\times 10^{4\;\;}{\hat{u}}_{1}+2.275\times 10^{6\;}{\hat{u}}_{2}+22.2\times 10^{5\;}{\hat{u}}_{3}$
$S_{6}\to {\vec{C}}_{6}$	$-6.68\times 10^{4\;\;}{\hat{u}}_{1}$ + 2.41×10^5^ ${\hat{u}}_{2}$ + 177608 ${\hat{u}}_{3}$	$-8.35\times 10^{5\;\;}{\hat{u}}_{1}+3.021\times 10^{6\;}{\hat{u}}_{2}+22.2\times 10^{5\;}{\hat{u}}_{3}$
$S_{7}\to {\vec{C}}_{7}$	$-2.21\times 10^{4}{\hat{u}}_{1}$ + 1.978×10^5^ ${\hat{u}}_{2}$ + 177608 ${\hat{u}}_{3}$	$-2.77\times 10^{5\;\;}{\hat{u}}_{1}+2.472\times 10^{6\;}{\hat{u}}_{2}+22.2\times 10^{5\;}{\hat{u}}_{3}$
$S_{8}\to {\vec{C}}_{8}$	$-2.44\times 10^{4}{\hat{u}}_{1}$ + 1.979×10^5^ ${\hat{u}}_{2}$ + 177608 ${\hat{u}}_{3}$	$-3.05\times 10^{5\;\;}{\hat{u}}_{1}+2.474\times 10^{6}\;{\hat{u}}_{2}+22.2\times 10^{5\;}{\hat{u}}_{3}$
$S_{9}\to {\vec{C}}_{9}$	$-2.65\times 10^{4\;}\;{\hat{u}}_{1}$ + 2.02×10^5^ ${\hat{u}}_{2}$ + 177608 ${\hat{u}}_{3}$	$-3.32\times 10^{5\;\;}{\hat{u}}_{1}+2.529\times 10^{6\;}{\hat{u}}_{2}+22.2\times 10^{5}{\hat{u}}_{3}$
$S_{10}\to {\vec{C}}_{10}$	$-2.97\times 10^{4\;}{\hat{u}}_{1}$ + 2.04×10^5^ ${\hat{u}}_{2}$ + 177608 ${\hat{u}}_{3}$	$-3.72\times 10^{5\;\;}{\hat{u}}_{1}+2.562\times 10^{6\;}{\hat{u}}_{2}+22.2\times 10^{5}{\hat{u}}_{3}$

**Table 2:** Similarity measurement between vectors in the texture space, cosφ when λ = 2.

Experimental Results for λ = 2 (First Confusion Matrix)											
**Tree stem images (prototypes)**											
**Tree stem images (test)**		1	2	3	4	5	6	7	8	9	10
	1	1.0000	0.9919	0.9453	0.9997	0.9985	0.9583	0.9923	0.9915	0.9898	0.9880
	2	0.9919	1.0000	0.9759	0.9916	0.9970	0.9868	0.9999	0.9999	0.9998	0.9996
	3	0.9453	0.9759	1.0000	0.9424	0.9576	0.9938	0.9745	0.9764	0.9780	0.9803
	4	0.9997	0.9916	0.9424	1.0000	0.9986	0.9577	0.9922	0.9912	0.9896	0.9878
	5	0.9985	0.9970	0.9576	0.9986	1.0000	0.9714	0.9973	0.9968	0.9958	0.9945
	6	0.9583	0.9868	0.9938	0.9577	0.9714	1.0000	0.9861	0.9872	0.9890	0.9908
	7	0.9923	0.9999	0.9745	0.9922	0.9973	0.9861	1.0000	0.9999	0.9998	0.9995
	8	0.9915	0.9999	0.9764	0.9912	0.9968	0.9872	0.9999	1.0000	0.9999	0.9996
	9	0.9898	0.9998	0.9780	0.9896	0.9958	0.9890	0.9998	0.9999	1.0000	0.9999
	10	0.9880	0.9996	0.9803	0.9878	0.9945	0.9908	0.9995	0.9996	0.9999	1.0000

**Table 3:** Similarity measurement between vectors in the texture space, cosφ when λ = 25.

Experimental Results for λ = 25 (Second Confusion Matrix)											
**Tree stem images (prototypes)**											
**Tree stem images (test)**		1	2	3	4	5	6	7	8	9	10
	1	1.0000	0.9919	0.9453	0.9997	0.9985	0.9583	0.9923	0.9915	0.9898	0.9880
	2	0.9919	1.0000	0.9759	0.9916	0.9970	0.9868	0.9999	0.9999	0.9998	0.9996
	3	0.9453	0.9759	1.0000	0.9424	0.9576	0.9938	0.9745	0.9764	0.9780	0.9803
	4	0.9997	0.9916	0.9424	1.0000	0.9986	0.9577	0.9922	0.9912	0.9896	0.9878
	5	0.9985	0.9970	0.9576	0.9986	1.0000	0.9714	0.9973	0.9968	0.9958	0.9945
	6	0.9583	0.9868	0.9938	0.9577	0.9714	1.0000	0.9861	0.9872	0.9890	0.9908
	7	0.9923	0.9999	0.9745	0.9922	0.9973	0.9861	1.0000	0.9999	0.9998	0.9995
	8	0.9915	0.9999	0.9764	0.9912	0.9968	0.9872	0.9999	1.0000	0.9999	0.9996
	9	0.9898	0.9998	0.9780	0.9896	0.9958	0.9890	0.9998	0.9999	1.0000	0.9999
	10	0.9880	0.9996	0.9803	0.9878	0.9945	0.9908	0.9995	0.9996	0.9999	1.0000

**Table 4:** Confusion matrix obtained for the image classification when λ=2.

Experimental Results for λ = 2											
**Tree stem images (prototypes)**											
**Tree stem images (test)**		1	2	3	4	5	6	7	8	9	10
	1	1	0	0	0	0	0	0	0	0	0
	2	0	1	0	0	0	0	0	0	0	0
	3	0	0	1	0	0	0	0	0	0	0
	4	0	0	0	1	0	0	0	0	0	0
	5	0	0	0	0	1	0	0	0	0	0
	6	0	0	0	0	0	1	0	0	0	0
	7	0	0	0	0	0	0	1	0	0	0
	8	0	0	0	0	0	0	0	1	0	0
	9	0	0	0	0	0	0	0	0	1	0
	10	0	0	0	0	0	0	0	0	0	1

**Table 5:** Confusion matrix obtained for the image classification when λ=25.

Experimental Results for λ = 25											
**Tree stem images (prototypes)**											
**Tree stem images (test)**		1	2	3	4	5	6	7	8	9	10
	1	1	0	0	0	0	0	0	0	0	0
	2	0	1	0	0	0	0	0	0	0	0
	3	0	0	1	0	0	0	0	0	0	0
	4	0	0	0	1	0	0	0	0	0	0
	5	0	0	0	0	1	0	0	0	0	0
	6	0	0	0	0	0	1	0	0	0	0
	7	0	0	0	0	0	0	1	0	0	0
	8	0	0	0	0	0	0	0	1	0	0
	9	0	0	0	0	0	0	0	0	1	0
	10	0	0	0	0	0	0	0	0	0	1

## Data Availability

The data related to the results that support our conclusions are available upon request to the authors, which can be carried out via e-mail. We will be pleased to respond.
